# Biological Heterogeneity

**DOI:** 10.1093/geroni/igaa057.3145

**Published:** 2020-12-16

**Authors:** Blanka Rogina

**Affiliations:** UConn Health, Farmington, Connecticut, United States

## Abstract

Studies of aging in invertebrates, mammalian animal models and humans have demonstrated increasing heterogeneity with aging in terms of varied facets of biological aging. In addition to growing heterogeneity, aging is also associated with qualitative and quantitative changes involving DNA methylation captured in epigenetic clocks of aging which seek to predict chronological and biological aging. Increased heterogeneity with aging is also evident in terms of posttranslational histone modification, gene expression, somatic clonal expansion, and increased degree of tissue mosaicism. Senescent cells accumulating with aging demonstrate significant heterogeneity. For example, while most studies targeting senescent cells have focused on cells expressing p16 (CDKN2A), not all p16-positive cells are senescent and not all senescent cells express p16. Further studies are needed to better define heterogeneity involving other hallmarks of aging and to also explore associations between heterogeneity involving these biological measures with clinical manifestations or outcomes.

